# Magnet-responsive, superhydrophobic fabrics from waterborne, fluoride-free coatings†

**DOI:** 10.1039/c7ra10941e

**Published:** 2018-01-03

**Authors:** Sida Fu, Hua Zhou, Hongxia Wang, Jie Ding, Shuai Liu, Yan Zhao, Haitao Niu, Gregory C. Rutledge, Tong Lin

**Affiliations:** Institute for Frontier Materials, Deakin University Geelong VIC3216 Australia hong.wang@deakin.edu.au; Defence Science and Technology Group Fishermans Bend VIC 3207 Australia; School of Material and Electric Engineering, Soochow University 215000 China; Department of Chemical Engineering, Massachusetts Institute of Technology Cambridge MA 02139 USA

## Abstract

In this study, durable superhydrophobic fabrics with magnet responsive properties were prepared by a two-step coating technique using polydopamine (PDA), Fe_3_O_4_ nanoparticles, and hexadecyltrimethoxysilane as coating materials. The coated fabrics exhibit fast magnetic responsivity and a water contact angle of 156°. The coating is durable enough to withstand at least 50 cycles of home laundering and 500 cycles of Martindale abrasions without losing its superhydrophobicity and magnetic properties. The PDA pre-coating plays a significant role in improving the adhesion of hydrophobic Fe_3_O_4_ nanoparticles on fabric surface. The coated fabric is highly oleophilic (oil contact angle = 0°). When used for absorbing oil, the coated fabric floats naturally on the surface of oily water, and it can be moved to approach oil drops under magnetic actuation. The fabric is reusable for at least 10 cycles. This may offer an environmentally friendly way to prepare “smart” oil-recovery materials.

## Abstract 2

Durable superhydrophobic fabrics with magnetic response ability have been prepared by waterborne, fluoride-free coatings.

## Graphical abstract

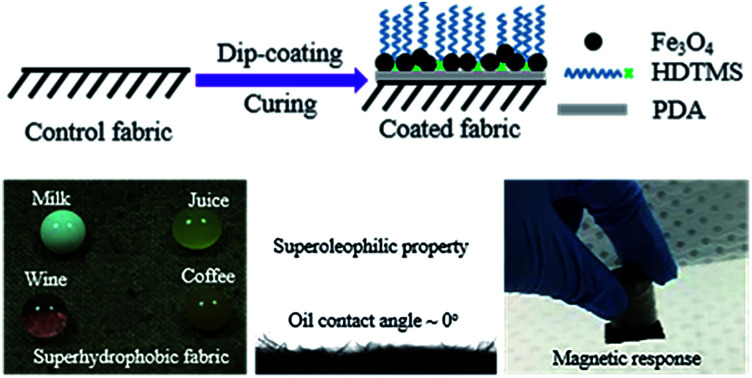

## 1. Introduction

Superhydrophobic surfaces have shown wide applications in anti-corrosion,^1^ oil/water separation,^2^ drag-reduction,^3^ self-cleaning,^4^ anti-fogging,^5^ and anti-icing.^6^ They can be fabricated by forming a nano-/microstructure with low surface energy on substrates. Several methods have been developed to prepare superhydrophobic surfaces, using methods such as self-assembly,^7^ electrospinning,^8^ vapor deposition,^9^ plasma treatment,^10^ etching,^11^ lithography,^12^ or wet-chemical coating.^13^ However, most of the wet-chemical methods involve the use of organic solvents, which not only increase cost but also cause safety and environmental issues. Fluorinated chemicals, especially for those with perfluoroalkyl chain length longer than eight carbons, are another concern due to their potential in accumulation within human body and high price.^14–16^ Development of fluoride-free materials for superhydrophobic treatment is highly desirable. Some papers have reported the preparation of superhydrophobic surfaces using fluoride-free materials in solvent-borne solutions. For example, polydimethylsiloxane (PDMS)–tetrahydrofuran (THF) solutions containing silica aerogel^17^ or onion-like carbon microspheres,^18^ SiO_2_-containing polystyrene solution in THF,^19^ and poly(styrene-methyl methacrylate-acrylic acid) nanospheres/polyacrylate/carbon black in acetone^20^ have been reported. Recently, fluorine-free coatings from aqueous solutions were also developed for preparation of superhydrophobic fabrics. Wang *et al.*^21^ reported a superhydrophobic fabric prepared by coating aqueous dopamine solution containing hexadecyltrimethoxysilane on fabric surface.

Superhydrophobic porous materials with an oleophilic surface have potential applications in oil–water separation and recovery of oil from water. When an oil–water mixture contacts with a superhydrophobic–oleophilic surface, water is repelled, whereas oil selectively spreads on the surface.^22^ Fabrics and sponges with a superhydrophobic–oleophilic surface selectively absorb oil from water with a much larger absorption capability than a flat surface. Gu *et al.*^23^ reported a polylactic acid nonwoven fabric with superhydrophobic–oleophilic property that could effectively absorb oil from water, even if the fabric was rigorously abraded or stretched. Diao *et al.*^24^ prepared superhydrophobic polystyrene (PS)/nonwoven fabric that is able to absorb both light oil and heavy oil. Cao *et al.*^25^ used nanodiamond particles to modify polyurethane (PU) sponge, and after modification the sponge showed strong capacity to absorb oil. Li *et al.*^26^ reported a candle soot-SiO_2_-modified PU sponge, which not only can absorb a series of oils, but also withstand a variety of harsh treatments.

Recently, magnetic properties were reported to combine with superhydrophobicity for oil absorption. The introduction of magnetic responsiveness was expected to simplify the recovery of the oil absorber and reduce the oil leakage from the absorber during collection with mechanical equipment. Zhang *et al.*^27^ fabricated a magnetic PU sponge with hierarchical structure based on a breath figure method. Driven by a magnetic field in polluted water, the sponge showed high absorption capacity to various oils. Lu *et al.*^28^ prepared superhydrophobic magnetic ethyl cellulose sponges by a freeze-drying method. The sponges were divided into small pieces and dispersed into water for oil absorption. After absorption, the small pieces of oil absorber were collected by a magnetic bar. However, little has been reported about the durability of magnetic, superhydrophobic oil absorbers.

In this study, we prepared a magnetic, superhydrophobic fabric using a two-step coating treatment. A thin layer of polydopamine (PDA) was pre-applied onto fabric substrate, followed by surface application of hydrophobic Fe_3_O_4_ nanoparticles. The coated fabric showed a water contact angle of 156° and a sliding angle of 5°, with a superoleophilic surface, allowing it to be used as an oil absorbent. We showed that the presence of PDA pre-coating significantly improved the adhesion of hydrophobic Fe_3_O_4_ nanoparticles to the fabric surface. The fabric was reusable for many cycles without losing this surface feature. Also, the magnetic property enables the coated fabric to approach oil drops under the actuation of a magnetic field.

## 2. Experimental section

### 2.1. Materials

Ammonium hydroxide (28% in water), dopamine hydrochloride, ferric(iii) chloride hexahydrate (FeCl_3_·6H_2_O), hexadecyltrimethoxysilane (HDTMS), chloroform, dichloromethane, tetrahydrofuran, hexadecane, and hydrochloric acid were obtained from Sigma-Aldrich. Ferrous(ii) chloride tetrahydrate (FeCl_2_·4H_2_O) was obtained from Merck. All chemicals were used as received. Commercial cotton fabric (plain weave, 165 g m^−2^, thickness ≈ 460 μm), polyester fabric (plain weave, 168 g m^−2^, thickness ≈ 520 μm), and wool fabric (plain weave, 190 g m^−2^, thickness ≈ 540 μm) were purchased from a local textile shop. The magnetic bar and soybean oil were purchased from a local supermarket. Gasoline was obtained from a Shell service station.

### 2.2. Coating of PDA on fabric

Dopamine hydrochloride (0.8 g) was added into deionized water (400 mL) and stirred for 5 min. Then, the pH of the mixture was adjusted to 8.5 with ammonium hydroxide, and a piece of cotton fabric (5 cm × 15 cm) was added into the resultant solution followed by stirring for 16 hours at room temperature. The resulting brown fabric was rinsed with water, and finally dried at 120 °C for 1 hour.

### 2.3. Preparation of magnetic, superhydrophobic fabrics

4 g HDTMS was added into 40 mL deionized water followed by adjusting pH value to 1 using hydrochloric acid, and then stirring for 8 hours. After that, pH value of the mixture was tuned to 8 by ammonium hydroxide. Aqueous NH_4_OH solution (1.5 M) was added dropwise to 360 mL water solution containing FeCl_3_·6H_2_O (1.7 g) and FeCl_2_·4H_2_O (0.60 g), followed by vigorous stirring under nitrogen protection.^29^ The solution was then mixed with the HDTMS solution. The solution mixture was applied onto the PDA coated fabric using a dip-coating method. After stirring at 60 °C for 16 hours, the resulting fabric was rinsed with water, and dried at 140 °C for 1 hour.

### 2.4. Washing durability test

Washing durability of the coated fabrics was tested by reference to the washing procedure specified in the AATCC (American Association of Textile Chemists and Colorists) Test Method 61-2006 test no. 2A. In short, washing solution was prepared by adding 0.225 g detergent in 150 mL water. The sample (5 cm × 15 cm) and 50 steel balls were added to the washing solution. The test was performed using a standard washing machine (MODEL H-240, NO. 4361, RAPID LABORTEX CO., LTD.). After running at 40 ± 2 rpm for 45 min at 49 °C, the sample was rinsed three times with distilled water and dried at room temperature. This standard washing procedure is equivalent to five cycles of home laundering.

### 2.5. Abrasion resistance test

The abrasion resistance of the superhydrophobic coating was evaluated using the Martindale method according to American Society for Testing and Materials (ASTM) D4966 test method, which is often used to evaluate heavy duty coated fabrics. The process was performed using a commercial Martindale abrasion tester (I.D.M Instrument Design & Maintenance). The test procedure should be in the standard atmosphere, which was 21 ± 1 °C and 65 ± 2%, and the load on the fabrics was 9 kPa.

### 2.6. Oil absorption capacity and reusable tests

A piece of coated fabric was soaked in various oily liquids for 5 seconds, and then taken out and drained for several seconds to remove excess oil. The weight of fabric before and after absorption was recorded. It was then washed with tap water containing dishwashing liquid three times, and dried in an oven at 80 °C. The oil absorption capacity *R* (g g^−1^) was determined by weighing the fabric before and after oil absorption according to the equation:*R* = (*M*_2_ − *M*_1_)/*M*_1_where *M*_1_ and *M*_2_ are the weight of the fabric before and after oil absorption.

### 2.7. Other characterizations

Scanning electron microscope (SEM) images were taken using a Jeol Neoscope SEM operated at an acceleration voltage of 10.0 kV. The contact angle (CA) was measured using a Contact Angle Meter (KSV Model CAM 101) with liquid droplets about 3 μL in volume. All the CA values were the average of 6 measurements. Sliding angles were measured using a purpose-made device consisting of a sample holder and a digital angle meter. Fourier Transform Infrared Spectroscopy (FTIR) spectra were measured using a Burker Vetex 70 instrument in Attenuated Total Reflection (ATR) mode. The spectra were obtained under 64 scans at 4 cm^−1^ resolution. X-ray photoelectron spectroscopy (XPS) spectra were recorded on a VG ESCALAB 220-iXL XPS spectrometer with a monochromated AL Kα source (1486.6 eV) using samples of about 3 mm^2^ in size. The X-ray beam incidence angle is 0° with respect to the surface normal, which corresponds to a sampling depth of ≈10 nm. The collected XPS results were analyzed by the CasaXPS software. The transmittance of the fabric was measured on an Agilent Cary 5000 UV-vis-NIR spectrophotometer in the range of 200 to 800 nm. Magnetic properties were tested using a Quantum Design MPMS-5 direct-current superconducting quantum interference device (DC-SQUID) magnetometer. The magnetic field intensity was tested by a PASCO magnetic field sensor PS-2162. Transmission electron microscopy (TEM) images were acquired by a FEI Tecnai G-20 microscope.

## 3. Results and discussion

**Fig. 1:**
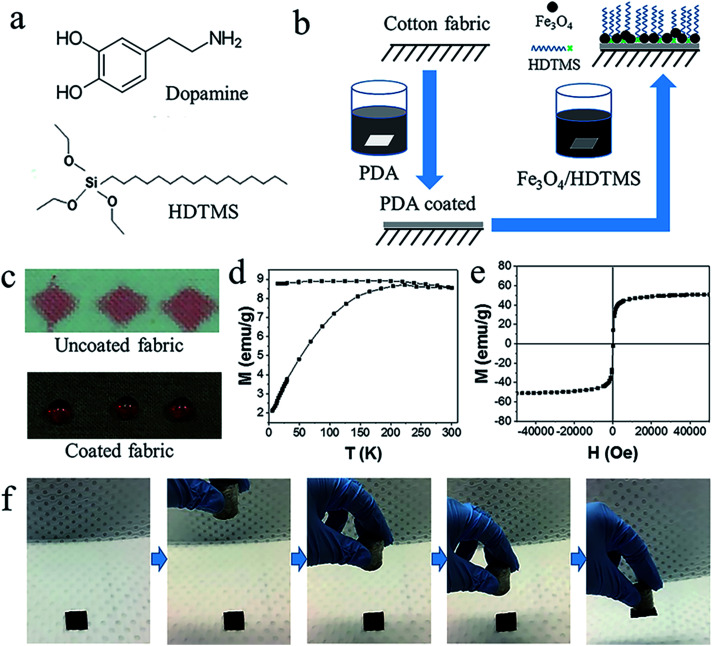
(a) Chemical structures of dopamine and HDTMS. (b) Schematic illustration of the procedure for preparing superhydrophobic cotton fabric. (c) Photos of red-colored water on the uncoated and coated cotton fabrics (10 μL for each drop; the small amount of dye used had no influence on the contact angle). (d) Zero field cooled-field cooled (ZFC-FC) curves measured with the field of 100 Oe. (e) Magnetization curve measured at 300 K. (f) Photos to show magnet response of the coated cotton fabric.

Fig. 1a shows the chemical structures of dopamine and HDTMS. Fig. 1b schematically illustrates the procedure to prepare magnetic superhydrophobic fabric. A two-step coating process was employed, in which PDA and Fe_3_O_4_/HDTMS were applied onto fabric in sequence. In the first step, a thin layer of PDA was applied onto the fiber surface. After that, Fe_3_O_4_ and HDTMS were co-deposited on the PDA surface. Both treatments were performed in aqueous solutions. After coating, the fabric color changed from white to brown, due to the dark color of PDA and Fe_3_O_4_. Fig. S1† shows the influence of the dark coating on the optical property. The coated fabric showed lower optical transmittance in the UV-vis region (wavelength 200–800 nm). This excellent UV shielding property may find applications in the UV-protection field.

Fig. 1c shows red-colored water droplets (10 μL) on the coated cotton fabric. Without coating treatment, cotton fabric was superhydrophilic. After coating treatment the fabric showed strong repellent to water, and water on the fabric formed sphere-like droplets. The coated fabric had a water contact angle (CA) of 156° and a sliding angle (SA) of 5°. To prove the stability of the water repellency, a water droplet (3 μL) was left on the coated fabric. As expected, the CA did not reduce until the droplet evaporated (Fig. S2†). The coated fabric also had high repellency to milk, juice, wine, and coffee. In addition, the fabric after coating treatment showed strong oleophilicity with an oil (soybean oil, 31.5 mN m^−1^) CA of 0°. When oil droplets contacted with fabric, they were spreading out immediately.

The magnetic properties of the as-prepared Fe_3_O_4_ nanoparticles were examined. Fig. 1d shows the ZFC-FC curves of the Fe_3_O_4_ nanoparticles. The blocking temperature was 220 K. Fig. 1e shows a typical superparamagnetic characteristic curve with no hysteresis appearing after removal of the applied magnetic field. At 300 K, the saturation magnetization is 50.81 emu g^−1^ at 5 T. When the Fe_3_O_4_ nanoparticles were applied onto fabric, a small piece of the coated fabric (2 cm × 2 cm) could be attracted by a magnet (Fig. 1f).

**Fig. 2:**
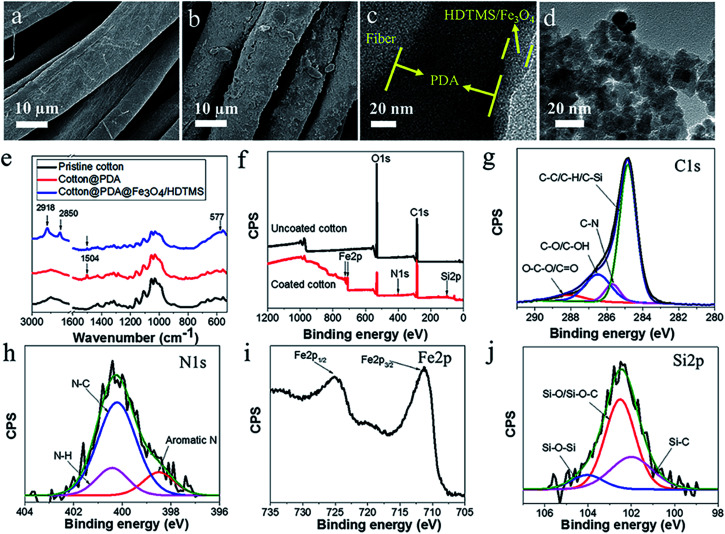
SEM images of cotton fibers (a) uncoated, and (b) PDA/Fe_3_O_4_/HDTMS coated. TEM images of (c) cross-section of the coating thickness and (d) Fe_3_O_4_ particles. (e) FTIR spectra and (f) XPS wide-survey spectra of cotton fabric before and after coating treatment. High-resolution XPS spectra of (g) C 1s, (h) N 1s, (i) Fe 2p, and (j) Si 2p of coated cotton fabric.

Fig. 2a and b show the SEM images of the fabric before and after PDA/HDTMS/Fe_3_O_4_ coating treatment. The uncoated fiber had a smooth surface (Fig. 2a). When the fiber was treated with PDA, the surface became rough (Fig. S3†). The surface roughness increased slightly after further coating with Fe_3_O_4_/HDTMS (Fig. 2b). The thicknesses of PDA and HDTMS/Fe_3_O_4_ coatings measured based on the TEM image were about 80 nm and 20 nm, respectively (Fig. 2c). The average size of Fe_3_O_4_ nanoparticles was about 10 nm (Fig. 2d).

FTIR and XPS were used to examine the surface chemistry of the fabric before and after coating treatment. As depicted in Fig. 2e, after PDA treatment, a new peak at 1504 cm^−1^ occurred, which was assigned to the N–H bending vibration, indicating the presence of PDA on the fabric surface.^30^ After coating with Fe_3_O_4_/HDTMS, new peaks at 2918 cm^−1^ and 2850 cm^−1^ appeared, corresponding to the asymmetric and symmetric vibrations of methylene (–CH_2_–). This indicates that long alkyl chains attach to the surface. A weak peak was observed at 577 cm^−1^, corresponding to the vibration of Fe–O–Fe bonds from Fe_3_O_4_.

Fig. 2f shows the XPS survey spectra of the fabrics. The presence of elements N, Fe, and Si confirmed that PDA and Fe_3_O_4_/HTDMS existed on the fiber surface. The quantitative element analysis results are presented in Table S1.† The atomic contents of the elements N, Fe, and Si in the coating surface were 0.65%, 2.17%, and 7.96%, respectively. The curved-fitted high-resolution C 1s spectra showed that the uncoated fabric had three peaks at 288.0 eV, 286.5 eV and 284.8 eV, corresponding to O–C–O/C=O, C–O/C–OH, and C–H/C–C, respectively (Fig. S4 and Table S2†). After coating treatment, two new peaks appeared at 285.7 eV and 284.8 eV, assigned to C–N and C–Si, respectively (Fig. 2g and Table S3†). The high-resolution N 1s, Fe 2p, and Si 2p spectra are shown in Fig. 2h–j. Three N 1s peaks at 400.4 eV, 400.2 eV, and 398.5 eV correspond to N–H, N–C and aromatic N moieties, respectively (Fig. 2h). The binding energies at 725 eV and 711 eV are characteristic of Fe 2p_1/2_ and Fe 2p_3/2_ of Fe_3_O_4_ (Fig. 2i). In addition, the three sub-peaks of Si 2p at 104.0 eV, 102.5 eV, and 102.0 eV corresponded to the Si–O–Si, Si–O/Si–O–C, and Si–C groups, respectively (Fig. 2j). These results suggest that the coating materials have applied to the fiber surface after coating treatment.

**Fig. 3:**
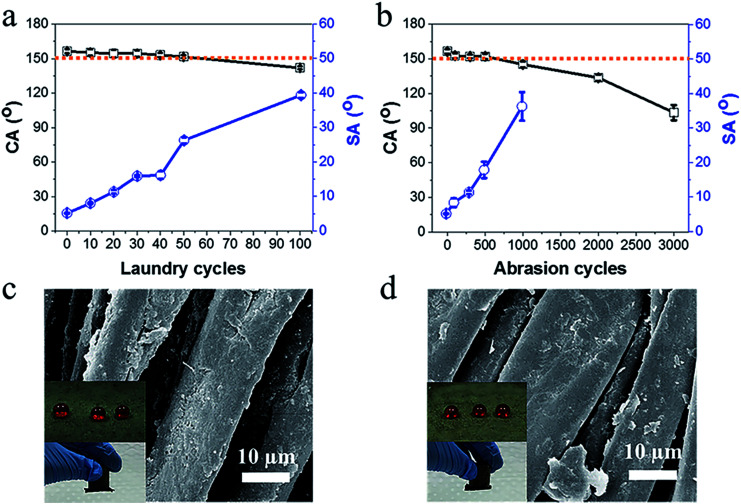
CA and SA of coated cotton fabrics change with (a) laundry cycles and (b) abrasion cycles. SEM images of coated fabrics (c) after 50 cycles of laundries and (d) 500 cycles of abrasions (the insert photos are water droplets on the coated cotton fabrics and a magnet attracting the fabrics).

Durability is an important criterion for superhydrophobic fabrics. Washing and abrasion durability of the coated fabrics were evaluated according to AATCC 61-2006 and ASTM D4966 standards. As shown in Fig. 3a, with an increase in the number of laundry cycles, the CA of the coated fabric decreased slightly, after 50 washing cycles, the fabric still maintained superhydrophobicity with a water CA of 152° and a SA of 26°. After 100 cycles of washing, the fabric was still hydrophobic with a CA of 142°, and a SA of 39.4°. The abrasion durability was tested using the Martindale method. To simulate actual wear, the load on the fabrics was set at 9 kPa. As shown in Fig. 3b, after 500 cycles of abrasions, contact angle only changed from 156° to 152°. Further increasing the abrasion cycles to 3000 led to a decrease of CA to about 103°.

Fig. 3c shows the SEM image of the fibers after 50 cycles of washing. The surface morphology was almost unchanged. Fig. 3d shows the abraded surface. After 500 abrasion cycles, certain particles were lost from the fiber surface. However, the surface morphology was still very rough. The FTIR results also verified that the characteristic peaks at 2918 cm^−1^, 2850 cm^−1^, 1504 cm^−1^ and 577 cm^−1^ were still obvious (Fig. S5†). Moreover, the coated fabric still had magnetic properties after washing and abrasion (Table S4,† the insert photos in Fig. 3c and d).

**Fig. 4:**
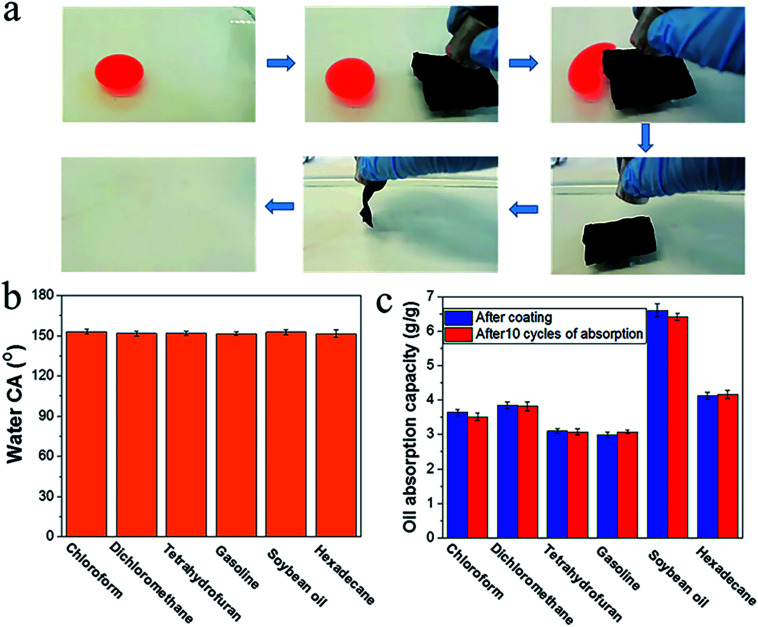
(a) Removal of hexadecane from water surface using superhydrophobic cotton fabric (hexadecane (1 mL) was coloured with oil red for clear observation, fabric (5 cm × 5 cm)). (b) Water CA and (c) oil absorption capacities for the six kinds of oily liquids of the fabric after 10 cycles of absorption–desorption.

Porous materials with a superhydrophobic and superoleophilic surface offer applications in oil recovery. When the materials also have a magnetic property, they can be positioned through an external magnetic field. To prove this, we used a piece of our superhydrophobic fabric to recover hexadecane in water. Upon placing the coated fabric in hexadecane polluted water, the fabric floated on the water surface. Actuated by a magnetic bar, the fabric can be moved to approach the oil contaminated area. Once oil drops attached to the fabric, they were absorbed rapidly into the fabric matrix. In this way, the oil on the water surface was cleaned completely by a small piece of fabric (Fig. 4a, also see Video 1 in the ESI†).

The fabric was able to be reused for oil recovery. After 10 cycles of absorption–desorption, the fabric maintained superhydrophobicity, and its oil absorption capacities for six types of oily liquids were almost unchanged (Fig. 4b and c).

Fig. S6† shows the possible route of dopamine polymerization.^31–34^ Dopamine can self-polymerize into PDA, which forms a thin coating layer on the substrate and it can further undergo secondary reactions like grafting of polymers, nanoparticles, and other functional molecules.^35–38^ In our case, hydrolyzed HDTMS can interact with Fe_3_O_4_ nanoparticles and catechol groups on PDA. Fe_3_O_4_ nanoparticles give the fabric a magnetic property, and HDTMS lowers its surface energy. Moreover, PDA can immobilize Fe_3_O_4_ nanoparticles and HDTMS on the surface of fiber, thus improving durability. The mechanism of Fe_3_O_4_/HDTMS coating on PDA modified fiber is illustrated in Fig. S7.†

To verify the role of PDA in the surface coating, we applied Fe_3_O_4_/HDTMS on the fabric substrate without pre-coating with PDA. After 10 cycles of washing and 100 cycles of abrasions, the fabric lost its superhydrophobicity (Fig. S8†). It was obvious that the surface morphology of fibers after washing and abrasion became smooth. We also tested the use performance of the Fe_3_O_4_/HDTMS coated fabric (without PDA pre-coating). As expected, after 10 cycles of absorption–desorption tests, the fabric lost both superhydrophobicity and magnetic response (Table S4†), though the oil absorption capacity remained, to a certain extent (Fig. S9†). The PDA per-coating largely improves adhesion of Fe_3_O_4_/HDTMS on the fiber surface.

It was noted that the HDTMS concentration affected the hydrophobicity of the coated fabric. Fig. S10† shows the effect of HDTMS concentration on the fabric. The water CA increased from 134° to 155° and SA reduced from 20.4° to 6.9°, with an increase in the HDTMS concentration from 0.1% to 1%. Furthermore, when the concentration changes from 1% to 4%, the fabric maintained its super-repellency. In contrast, the concentration of Fe_3_O_4_ nanoparticles has little effect on wetting property (Fig. S10†). However, when weight ratio is 1/10 (reference concentration is what we used in the above experiment), the fabric cannot be attracted by a magnet. When the weight ratio increased to 1/2, the fabric has a magnetic response.

Since PDA is able to adhere on virtually all kinds of inorganic and organic surfaces through a spontaneous polymerization reaction,^34^ pre-coating PDA followed by coating with Fe_3_O_4_/HDTMS should be a general method to prepare magnetic superhydrophobic fabrics. To verify this, we used the same method to treat polyester and wool fabrics. After PDA and Fe_3_O_4_/HDTMS coating, both polyester and wool fabrics turned superhydrophobic with magnet responsive property, and the coating showed high washing durability as well (Table S5†).

## 4. Conclusions

We have prepared a magnetic superhydrophobic fabric using waterborne, fluorine-free coating solutions. The coating treatment is free of organic solvent and fluoride, hence environmentally friendly. The coating is durable enough to withstand at least 50 cycles of home washing and 500 cycles of Martindale abrasions without losing its superhydrophobicity and magnetic property. The PDA pre-coating plays a significant role in improving the adhesion of hydrophobic Fe_3_O_4_ nanoparticles on fabric surface. The coated fabric is highly oleophilic (oil contact angle = 0°). When used for absorbing oil, the coated fabric floats naturally on the surface of oily water, and it can be moved to approach oil drops under magnetic actuation. The fabric is reusable for at least 10 cycles. Such a magnet-responsive superhydrophobic fibrous material may be useful for oil recovery and water purification.

## Conflicts of interest

There are no conflicts to declare.

## Supplementary Material

RA-008-C7RA10941E-s001

RA-008-C7RA10941E-s002
