# Supplementary material for: Magnet-responsive, superhydrophobic fabrics from waterborne, fluoride-free coatings
Source: RSC Adv. 2018 Jan 3;8(2):717–23. doi: 10.1039/c7ra10941e (PMC9076852; doi:10.1039/c7ra10941e)
Supplement: RA-008-C7RA10941E-s001 [file RA-008-C7RA10941E-s001.pdf]

Electronic Supplementary Material (ESI) for RSC Advances.
This journal is © The Royal Society of Chemistry 2017

Magnet-responsive, Superhydrophobic Fabrics from Waterborne, Fluoride-free Coatings

Sida Fu[a], Hua Zhou[a], Hongxia Wang[a]*, Jie Ding[b], Shuai Liu[c], Yan Zhao[a], Haitao Niu[a], Gregory C Rutledge[d], Tong Lin[a]

a Institute for Frontier Materials, Deakin University, VIC 3216, Australia
b Defence Science and Technology group, Fishermans Bend VIC 3207, Australia
c School of Material and Electric Engineering, Soochow University, 215000, China
d Department of Chemical Engineering, Massachusetts Institute of Technology, Cambridge, MA 02139

*Corresponding author.

E-mail address: hong.wang@deakin.edu.au.

Electronic Supplementary Information

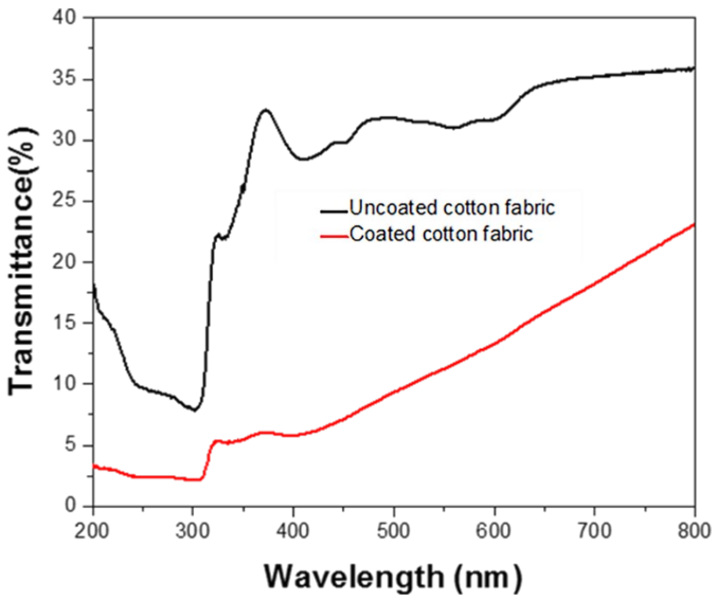

Fig. S1. UV-visible transmittance of uncoated and coated cotton fabrics.

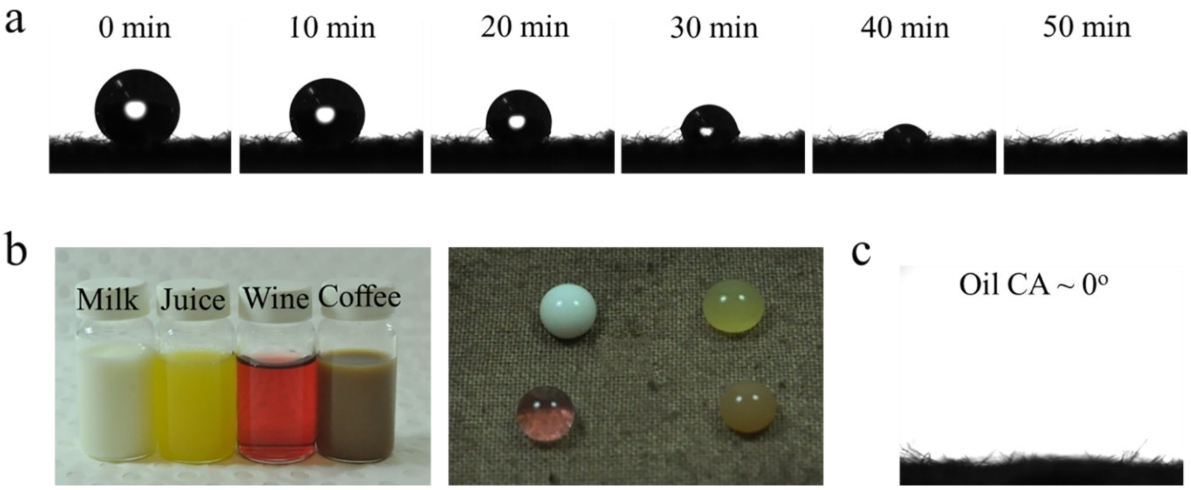

Fig. S2. a) The CA images of water droplet (~ 3 μL) with time. b) Milk, juice, wine, and coffee on the coated fabric. c) Oil CA of coated fabric.

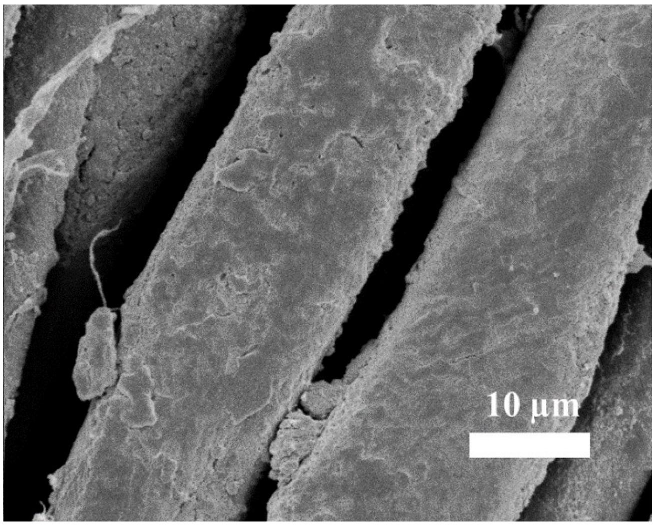

Fig. S3. SEM image of the fabric after PDA coating.

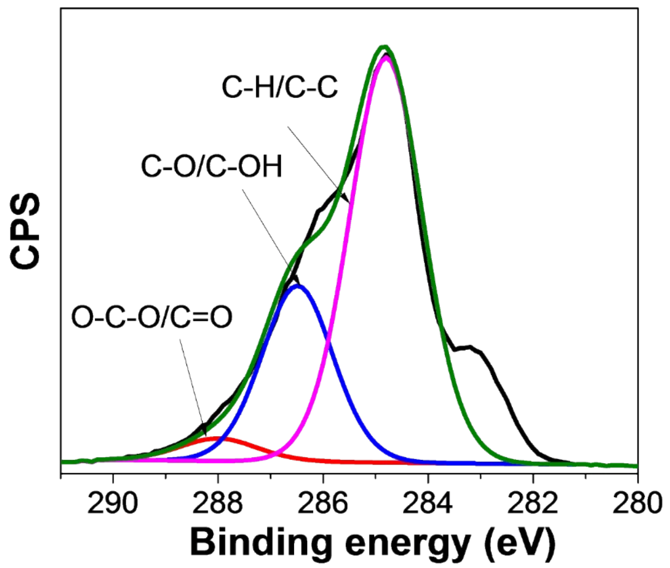

Fig. S4. High-resolution C1s spectra and curve fitted results of the uncoated fabric.

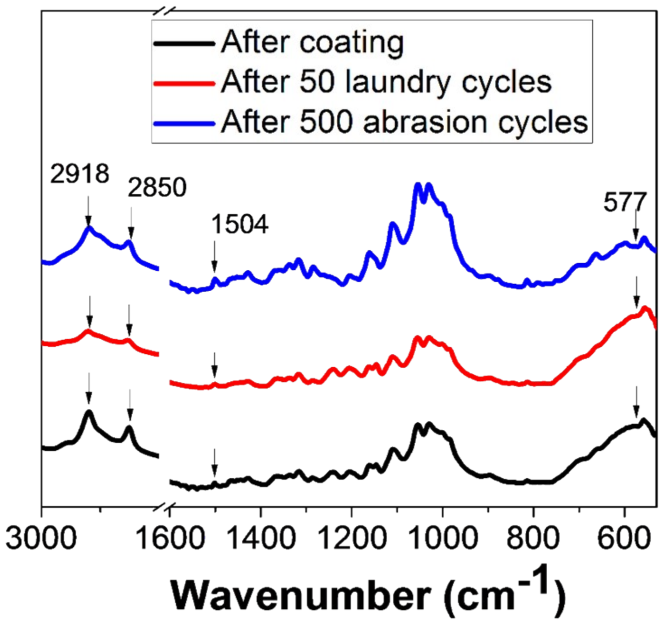

Fig. S5. FTIR spectra of coated fabric after 50 cycles of laundries and 500 cycles of abrasion.

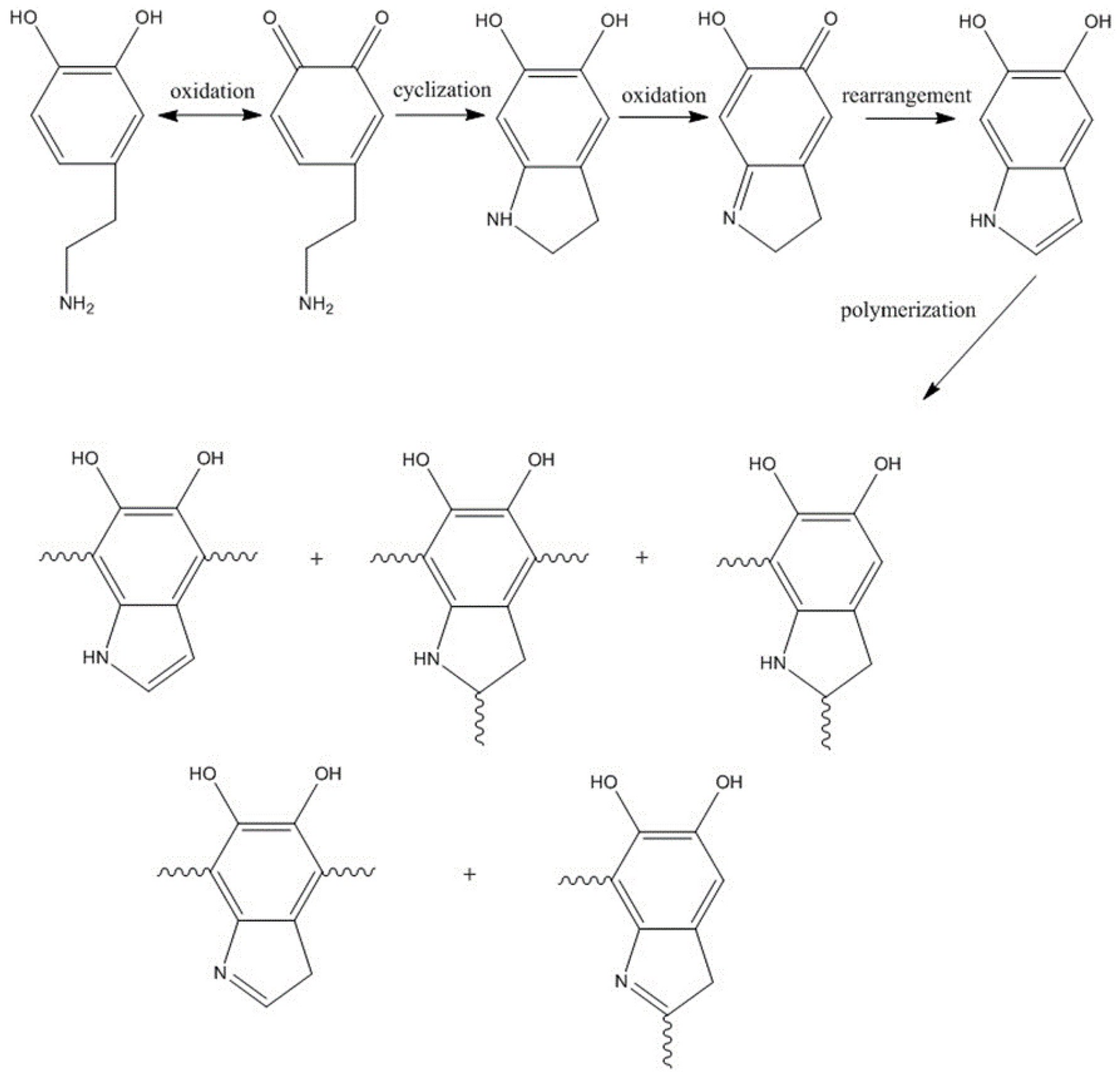

Fig. S6. Possible polymerization mechanism of dopamine.

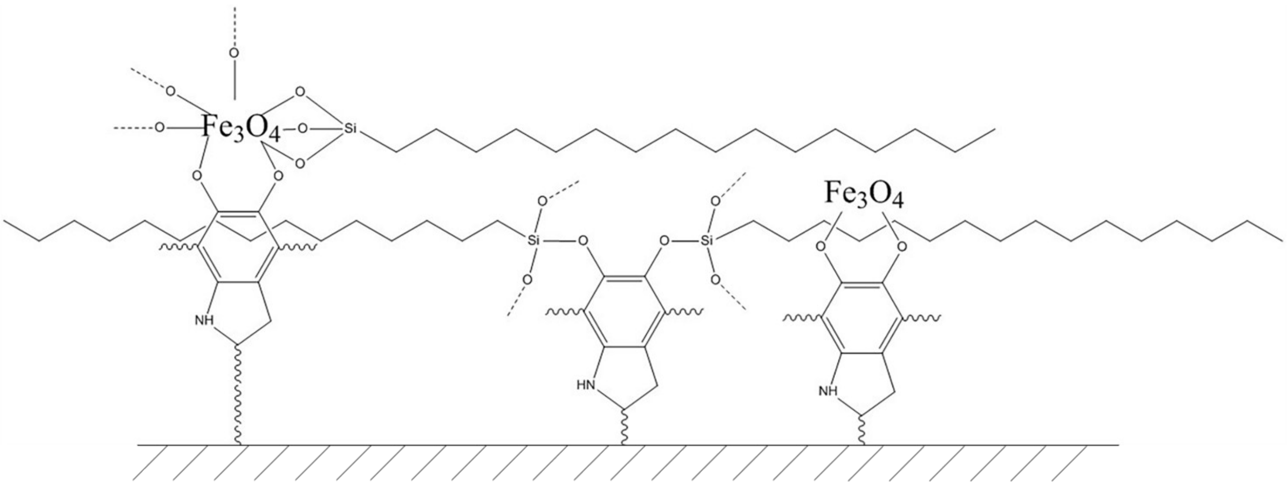

Fig. S7. Possible chemical reactions on the surface of PDA modified fiber.

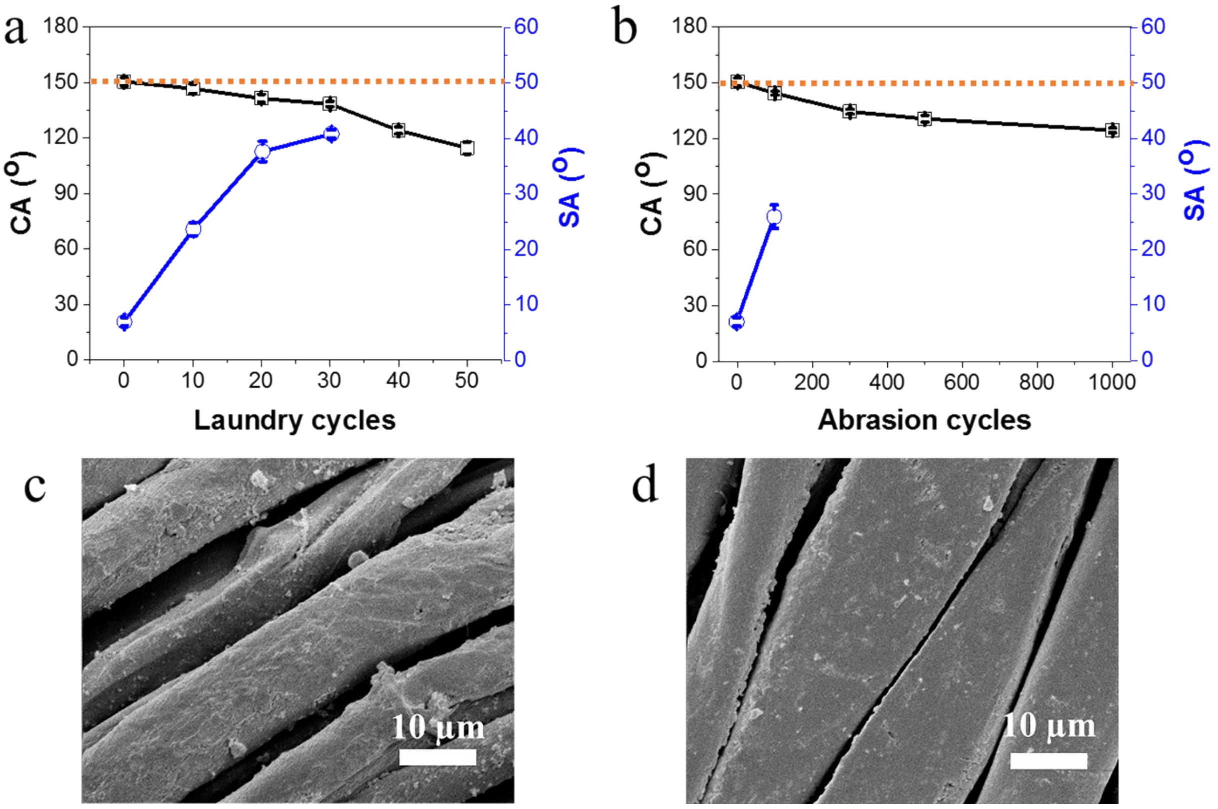

Fig. S8. CA and SA of cotton fabrics without PDA coating change with a) laundry cycles and b) abrasion cycles. SEM images of cotton fabrics without PDA coating c) after 50 cycles of laundries and d) 500 cycles of abrasion.

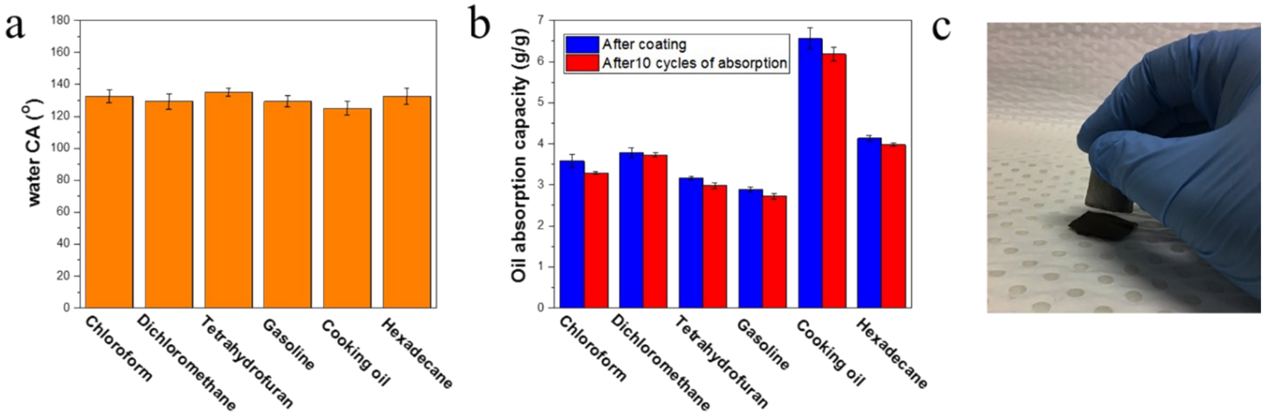

Fig. S9. a) Water CA and b) oil absorption capacities for the six kinds of oily liquids of the fabric (without PDA coating) after 10 cycles of absorption-desorption. c) The fabric cannot attract by magnet bar.

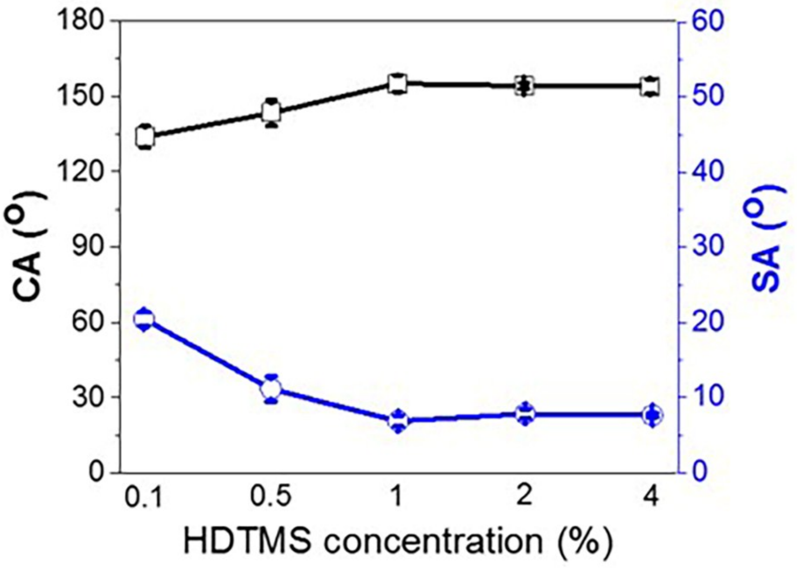

Fig. S10. The influence of the HDTMS concentration on hydrophobicity of cotton fabric.

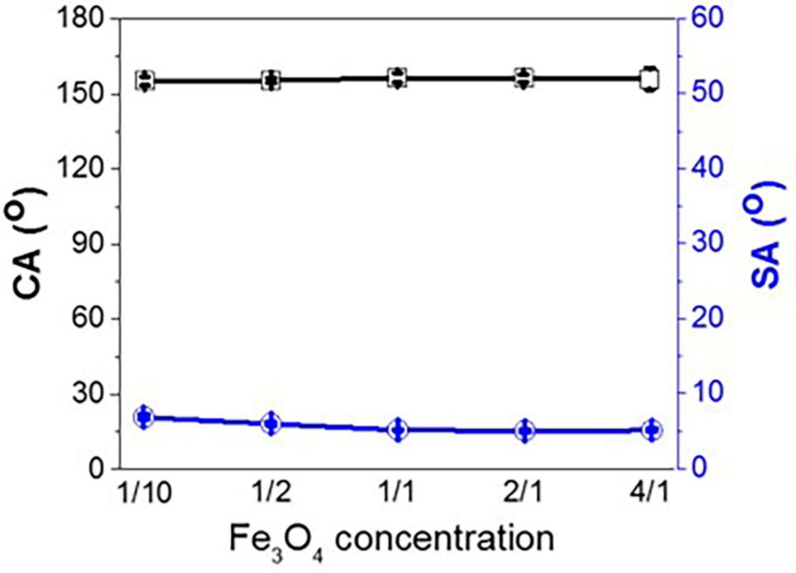

Fig. S11. The influence of the Fe_3O_4 concentration on wetting property (Reference concentration is what we used in the above experiment, HDTMS 1%).

Table S1. XPS spectrum parameters for the uncoated and coated cotton surfaces.

Sample	C1s (Atomic %)	O1s (Atomic %)	N1s (Atomic %)	Fe2p (Atomic %)	Si2p (Atomic %)
Uncoated	61.26	38.74	0	0	0
Coated	66.35	22.87	0.65	2.17	7.96

Table S2. Element contents (%) on the uncoated cotton surface.

	Peak	Binding energy (eV)	Atomic % (peak area)
C1s	O-C-O/C=O	288.0	4.00
	C-O/C-OH	286.5	28.46
	C-H/C-C	284.8	67.54

Table S3. Element contents (%) on the coated cotton surface.

	Peak	Binding energy (eV)	Atomic % (peak area)
C1s	O-C-O/C=O	288.1	5.41
	C-O/C-OH	286.5	20.2
	C-N	285.7	7.81
	C-C/C-H/C-Si	284.8	66.58
N1s	N-H	400.4	16.83
	N-C	400.2	68.99
	Aromatic N	398.5	14.18
Si2p	Si-O-Si	104.0	10.12
	Si-O/Si-O-C	102.5	59.9
	Si-C	102.0	29.98

Table S4. Magnetic response test.

	Magnetic response			
	After treatment	After 50 laundry cycles	After 500 abrasion cycles	After 10 cycles of repeated use
With PDA	Yes	Yes	Yes	Yes
Without PDA	Yes	No	No	No

Table S5. CA and SA of the coated cotton, wool and polyester fabrics before and after washing.

Fabric	After coating			After washing (50 cycles)		
	CA $(^{o})$	SA $(^{o})$	Photo	CA $(^{o})$	SA $(^{o})$	Photo
Cotton	156	5.1	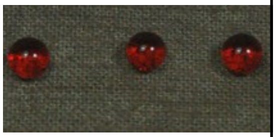	152	26.3	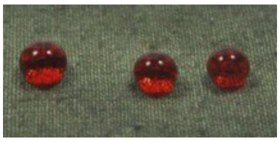
Wool	160	4.8	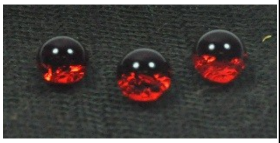	153	24.6	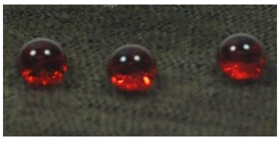
Polyester	154	6.9	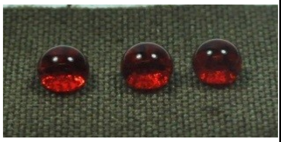	147	32.8	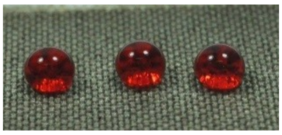

Video 1

Video 1 showed that a piece of coated fabric was driven by magnet to absorb hexadecane (oil red dyed) floated on water. The oil could be easily absorbed and then the fabric was picked up by magnet.
